# Ultralow-Barrier Antiferroelectric–Ferroelectric Transition Enabled by Competing Polar Distortion in Two-Dimensional Ruddlesden–Popper Nitride Perovskites

**DOI:** 10.1021/acs.nanolett.6c00448

**Published:** 2026-04-08

**Authors:** Shuyi Lin, Qiong Lei, Jun Yin

**Affiliations:** † Department of Applied Physics, Research Center for Organic Electronics, 26680 The Hong Kong Polytechnic University, Hung Hom, Kowloon, Hong Kong, 999077, China; ‡ Macao Institute of Materials Science and Engineering (MIMSE), Faculty of Innovation Engineering, 58816 Macau University of Science and Technology, Taipa, Macao 999078, China

**Keywords:** nitride perovskites, Ruddlesden−Popper phase, first-principles calculations, AFE−FE transition, polarization switching

## Abstract

Two-dimensional Ruddlesden–Popper (2D RP) nitride perovskites, combining strong covalency with reduced dimensionality and weak interlayer coupling, are promising candidates for low-energy polarization control. Here, we study 2D RP nitride perovskite La_2_WN_4_ using first-principles calculations in combination with symmetry analysis and mode decomposition. La_2_WN_4_ hosts a competing semiconducting ferroelectric (FE, *Aba*2) phase and antiferroelectric (AFE, *Pna*2_1_) phase, where polarization originates from WN_6_ octahedral distortions coupled to nitrogen displacements. Both the AFE–FE interconversion and polarization reversal proceed over low barriers, enabling fast, energy-efficient switching. External stimuli provide effective control, given that 1% biaxial compressive strain or an in-plane electric field of 0.025 V/Å can reversibly toggle between AFE and FE states. Moreover, La_2_WN_4_ can be exfoliated into a stable semiconducting monolayer that retains sizable in-plane polarization with a low switching barrier, highlighting its potential for low-power-programmable AFE/FE functionalities.

## Graphical abstract

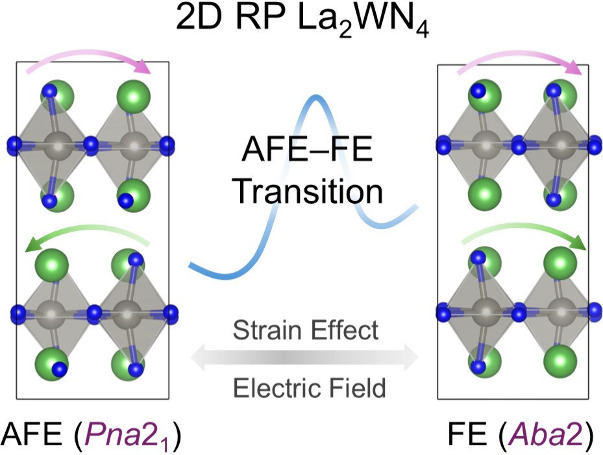

Ferroelectric (FE) and antiferroelectric (AFE) perovskites, characterized by switchable polarization, are promising for nonvolatile memories,^1–3^ high-energy-density capacitors,^4–6^ and spintronic devices.^7,8^ In conventional perovskites, a prototypical FE compound such as BaTiO_3_ develops a finite macroscopic polarization through spontaneous symmetry breaking,^9–11^ whereas a representative AFE compound like PbZrO_3_ exhibits a vanishing net polarization due to the antiparallel alignment of neighboring dipoles.^12–14^ Beyond the cubic ABX_3_ archetype, layered perovskites, especially A_n+1_B_n_X_3n+1_ Ruddlesden–Popper (RP) phases,^15–17^ combine strong intralayer bonding with weak interlayer coupling. This structural hierarchy enhances the interplay among lattice, orbital, and spin degrees of freedom and provides an intrinsically low-dimensional platform in which polarization can be efficiently manipulated by external stimuli, including electric field, strain, and even optical excitation.^18–21^

The weak interlayer coupling in RP phases can readily accommodate AFE arrangements, which may be driven into a FE state with a net polarization under applied fields, offering a route toward low-field polarization switching within a perovskite-compatible framework.^22,23^ However, realizing low-field, reversible AFE–FE transitions in practical materials remains challenging. First, phase switching often involves substantial lattice reconstruction, making it vulnerable to defect pinning, kinetic hysteresis, and fatigue.^24,25^ Second, strain engineering is constrained by substrate compatibility and the essentially static nature of epitaxial strain.^26–29^ Third, heterostructure-based designs frequently suffer from weak interfacial coupling and stringent requirements on structural matching during fabrication.^30–32^ These limitations motivate the search for new material platforms that feature strong electron–phonon coupling, intrinsically reduced AFE–FE energy barriers, and robust tunability under experimentally accessible external fields.

Nitride perovskites represent a particularly attractive family for enabling such functionalities. Compared with oxide perovskites, their enhanced covalency and higher cation oxidation states tend to promote pronounced lattice distortions and stronger electron–phonon coupling,^33,34^ thereby favoring the stabilization and controllability of polar order. Indeed, several nitride perovskites, such as ThTaN_3_ and LnMN_3_ (Ln = La, Ce; M = W, Re, Mo, Ta), have been predicted or synthesized as FE materials in recent years.^35–40^ Nevertheless, studies of RP-type nitrides such as Ce_2_TaN_4_ indicate that, while high-pressure synthesis can establish the structural feasibility of RP nitride perovskite phases, a ferromagnetic metallic state associated with Ce^3+^ (4*f* ^1^) can strongly screen the polarization field, suppressing FE/AFE order.^41^ These observations underscore the importance of identifying layered nitride RP phases that combine nonmagnetic 4*f* ^0^ A-site cations with high-valent *d* ^0^ transition-metal B sites and an intrinsically insulating or semiconducting electronic structure, thereby enabling stable polar phases and low-field polarization switching.

Here, we investigate the two-dimensional (2D) RP nitride perovskite La_2_WN_4_ as a *d* ^0^ layered perovskite model system composed of La^3+^ (4*f* ^0^) and W^6+^ (5*d* ^0^). Using first-principles calculations together with group-theoretical and mode-decomposition analyses, we find that RP-type La_2_WN_4_ hosts two competing polar phases (i.e., the AFE *Pna*2_1_ phase and the FE *Aba*2 phase). The energy barrier between these two phases is only ∼78 meV/f.u., enabling reversible switching under 1% biaxial strain or an in-plane electric field of 0.025 V/Å. Moreover, owing to its RP structure, La_2_WN_4_ can be exfoliated into a stable 2D monolayer that retains a large in-plane polarization and exhibits a low switching barrier. The present AFE–FE competition and low-barrier switching echo recent phase-transition behaviors reported in oxide perovskites and unconventional multiferroics,^42–46^ suggesting that related nitride systems may provide a promising platform for magnetoelectric functionalities and electrically programmable AFE/FE devices. These results establish La_2_WN_4_ as a compelling nitride RP perovskite for elucidating mode-competition-driven AFE–FE phase transitions and for achieving programmable polarization control in low-dimensional systems.

In *d* ^0^ nitride perovskites, the emergence of ferroelectricity is governed by a strong coupling between lattice distortions and the electronic structure. For example, in CeTaN_3_ and LaWN_3_, the cubic paraelectric *Pm*3̅*m* phase, featuring an *f* ^0^ A-site and *d* ^0^ B-site configuration, lowers their symmetry through cooperative octahedral distortion and polar cation displacements, thereby developing ferroelectric structures that remain semiconducting (Figures 1a, 1b). Consistent with this, the phonon dispersion for cubic LaWN_3_ exhibits pronounced imaginary branches at the M and R points (Figure 1c), indicating a dynamical instability associated with octahedral rotations and polar distortions; condensation of these unstable modes drives the transition into a low-symmetry ferroelectric phase.

**Figure 1:**
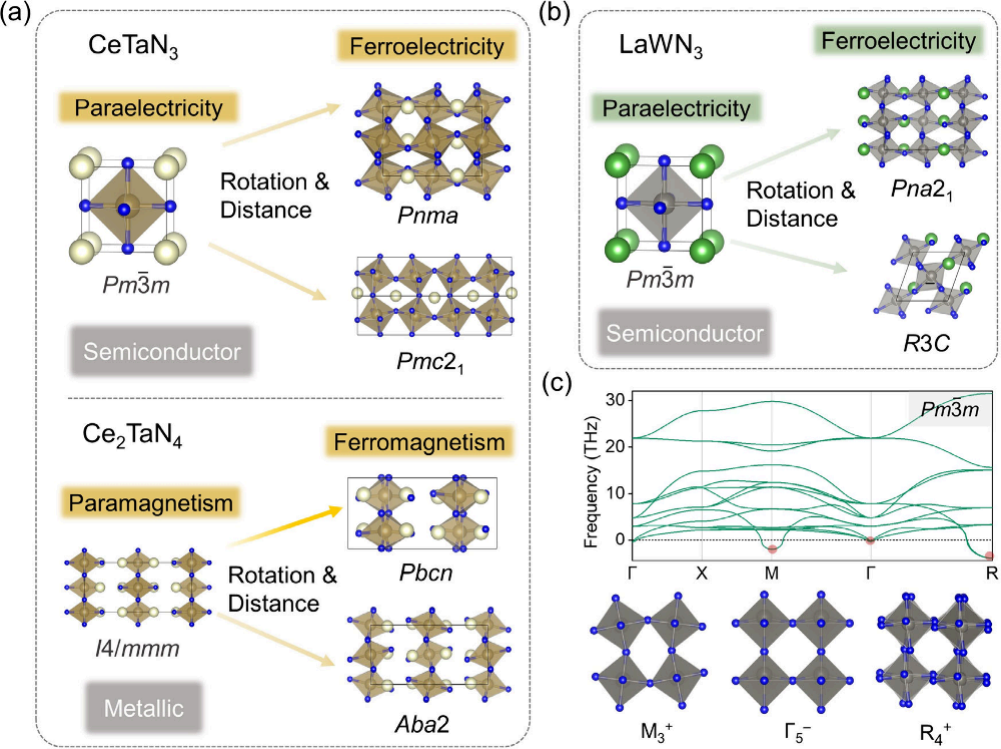
(**a**) Structural phase transitions in nitride lanthanide perovskites CeTaN_3_ and Ce_2_TaN_4_, showing the transformation from paraelectric phase (*Pm*3̅*m*) to ferroelectric phase (*Pnma* and *Pmc*2_1_) and from paramagnetic phase (*I*4*/mmm*) to ferromagnetic phase (*Pbcn* and *Aba*2), driven by octahedral rotation and polar distortions. (**b**) Structural phase transitions in nitride lanthanide perovskite LaWN_3_ from paraelectric phase (*Pm*3̅*m*) to ferroelectric phase (*Pna*2_1_ and *R*3*C*) via octahedral rotation and polar distortions. (**c**) Calculated phonon dispersion of LaWN_3_ in the cubic *Pm*3̅*m* phase along high-symmetry paths in the Brillouin zone: Γ (0 0 0) – X (0.5 0 0) – M (0.5 0.5 0) – Γ (0 0 0) – R (0.5 0.5 0.5). Imaginary phonon branches, particularly at the M and R points, indicate the dynamic instability associated with octahedral rotational modes.

By contrast, in the 2D RP nitride Ce_2_TaN_4_, the involvement of Ce at the A-site introduces an additional complication. Although the RP framework still supports sizable structural distortions, the compound becomes metallic, mainly due to contributions from the Ce-4*f* orbital electron (as shown in Figure S2), and the resulting free carriers effectively screen the polarization field. This screening suppresses long-range polar order and thus impedes the stabilization and experimentally observable ferroelectric state. To disentangle A-site effects from the intrinsic *d* ^0^-driven ferroelectric mechanism, we therefore focus on the 2D RP compound La_2_WN_4_, in which La^3+^ provides a nonmagnetic *f* ^0^ A-site while W^6+^ maintains a *d* ^0^ B-site, yielding an electronic structure that is favorable for robust polar ordering.

The emergence of a low-symmetry phase from a high-symmetry parent can be rationalized as a symmetry-breaking instability, as formalized within the isotropy subgroup theory. In this framework, the distortion patterns that connect the parent and distorted structures are expressed as basis functions of irreducible representations (irreps) of the parent space group and act as symmetry-adapted order parameters for the transition. In displacement-type transitions, the symmetry lowering is driven by collective atomic displacements away from high-symmetry positions, providing a direct atomistic description of how specific lattice modes generate the observed structural and functional changes. Guided by this approach, we systematically generated low-symmetry candidates for the 2D RP nitride La_2_WN_4_ starting from the high-symmetry *I*4/*mmm* prototype with intact WN_6_ octahedra (Figure 2a). We introduced the minimal set of irreps required to capture octahedral distortions in this RP framework, identifying *i*) an in-plane polar (ferroelectric) Γ_5_^–^ mode, *ii*) an octahedral tilt X_3_^+^ mode, and *iii*) two antiferroelectric modes, M_5_^–^ and M_2_^+^. The associated order parameter directions are (*a*, −*a*), (*a*, *b*), (0, *a*), and (*a*, −*a*), respectively. By calculating the phonon dispersion curves of the high-symmetry prototype phase, consistent possible structural distortion patterns are also obtained (Figure 2b). The negative frequencies indicated the instabilities of the prototype phase and displayed a much richer set of structural instabilities than those that correspond to host LaWN_3_ (Figure 1c). Notably, the Γ_5_^–^ and M_5_^–^ modes correspond to opposite displacement patterns and are expected to produce opposite polarization directions.

**Figure 2:**
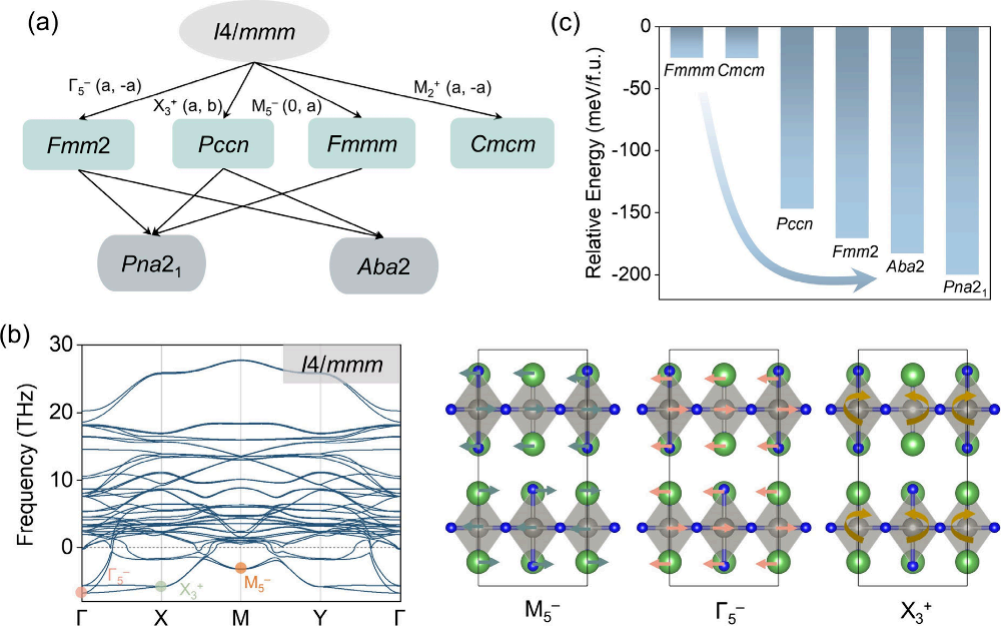
(**a**) Symmetry-adapted lattice-distortion modes of La_2_WN_4_ in the *Pna*2_1_ and *Aba*2 phases, derived from the group–subgroup relationship referenced to the high-symmetry *I*4/*mmm* parent structure. Connecting lines between space groups indicate that the symmetry-allowed transitions are continuous within Landau theory. (**b**) Calculated phonon dispersion of La_2_WN_4_ in the *I*4/*mmm* phase, together with the dominant atomic displacements associated with the indicated irreducible representations. The phonon dispersion is plotted along high-symmetry paths in the Brillouin zone: Γ (0 0 0) – X (0.5 0 0) – M (0.5 0.5 0) – Y (0 0.5 0) – Γ (0 0 0). Imaginary phonon branches, particularly near Γ, X, and M, reveal dynamic instability arising from polar or octahedral tilt modes. (**c**) Relative energies of candidate phases (*Fmmm, Cmcm, Pccn, Fmm*2, *Aba*2, and *Pna*2_1_) with respect to the *I*4/*mmm* reference phase.

By freezing in individual unstable modes and selected linear combinations, we obtained six low-symmetry candidates, which are *Fmmm, Cmcm, Pccn, Fmm*2, *Aba*2, and *Pna*2_1_. Total energy calculations referenced to the *I*4/*mmm* parent show that *Pna*2_1_ and *Aba*2 phases are the most energetically favorable, lying 200.08 and 183.00 meV/f.u. below the prototype, respectively (Figure 2c). Phonon calculations further confirm that both phases are dynamically stable with no imaginary modes across the Brillouin zone (Figure S3). *Ab initio* molecular dynamics (AIMD) simulations show that *Pna*2_1_ and *Aba*2 lattices remain structurally intact at 300 and 400 K over 10 ps without signs of structural degradation (Figure S4). Additionally, their elastic constants satisfy the Born stability criteria^47^ (Table S1), establishing mechanical stability. These results show that La_2_WN_4_ hosts two competing polar ground states (*Pna2*_1_ and *Aba*2) within the RP perovskite family.

We next assess the electronic properties of the two polar phases by calculating orbital-projected band structures using the HSE functional, including spin–orbit coupling (SOC) and Hubbard U corrections (Figures S5, S6). For both phases, SOC narrows the band gap, whereas the inclusion of U tends to widen it, consistent with enhanced localization of W-*d* states. At the HSE+U+SOC level, the *Pna*2_1_ and *Aba*2 phases exhibit band gaps of 1.50 and 1.59 eV, respectively. Beyond the gap magnitude, SOC induces a clear spin splitting along the high-symmetry Q–Z path, which is perpendicular to the polarization direction. Importantly, these qualitative features are insensitive to the specific computational level: the valence band maximum is dominated by N-*p* states, while the conduction band minimum is dominated by W-*d* states. The distorted WN_6_ octahedral network generates a strong crystal-field environment that reshapes the W-5*d* manifold and promotes substantial mixing between the nominal *t*_2g_ and *e*_g_ states in the conduction bands. This symmetry-breaking distortion alters the local coordination of W^6+^ and removes the inversion symmetry, thereby stabilizing robust FE or AFE polarization.

Figure 3a compares the crystal structures of the *Pna*2_1_ and *Aba*2 phases, highlighting the microscopic differences between their polarization configurations in La_2_WN_4_. In *Pna*2_1_, the layer-resolved polarization, arising from distortions of the WN_6_ octahedra together with cooperative shifts of coordinating N ions within the W–N layers, is arranged antiparallel along the *c*-axis. This cancellation yields a vanishing net macroscopic polarization consistent with an AFE ground state. In contrast, in *Aba*2 the polarization vectors are aligned uniformly in all layers, producing a net macroscopic polarization of 20.20 μC/cm^2^. To elucidate the microscopic origin of its polarization, we calculated the Born effective charges (BECs) and atomic dipole moments (Figure S7). The BECs of W and La atoms strongly deviate from their nominal valences (W = +6.89 *e*), indicative of the anomalous dynamic charges typical of *d* ^0^ transition-metal systems and reflecting strong covalency coupling as well as pronounced coupling between lattice displacements and polarization within the WN_6_ framework. Both types of N atoms exhibit negative BECs with magnitudes slightly exceeding 3 *e*, signaling a substantial charge redistribution during the FE distortion. Consistently, the dipole-moment analysis shows comparatively small contributions from La and N atoms, whereas W atoms exhibit the dominant dipole moment of 5.70 D, demonstrating that the FE to PE transition is primarily driven by the off-centering of W, with cooperative N displacements partially compensating the local polarization. Moreover, Bader charge (Table S2) and electron localization function (Figure S8) analyses confirm that W–N bonds exhibit both stronger ionicity and slightly greater covalent mixing than La–N bonds, consistent with the dominant role of W off-centering in the polarization response.

**Figure 3:**
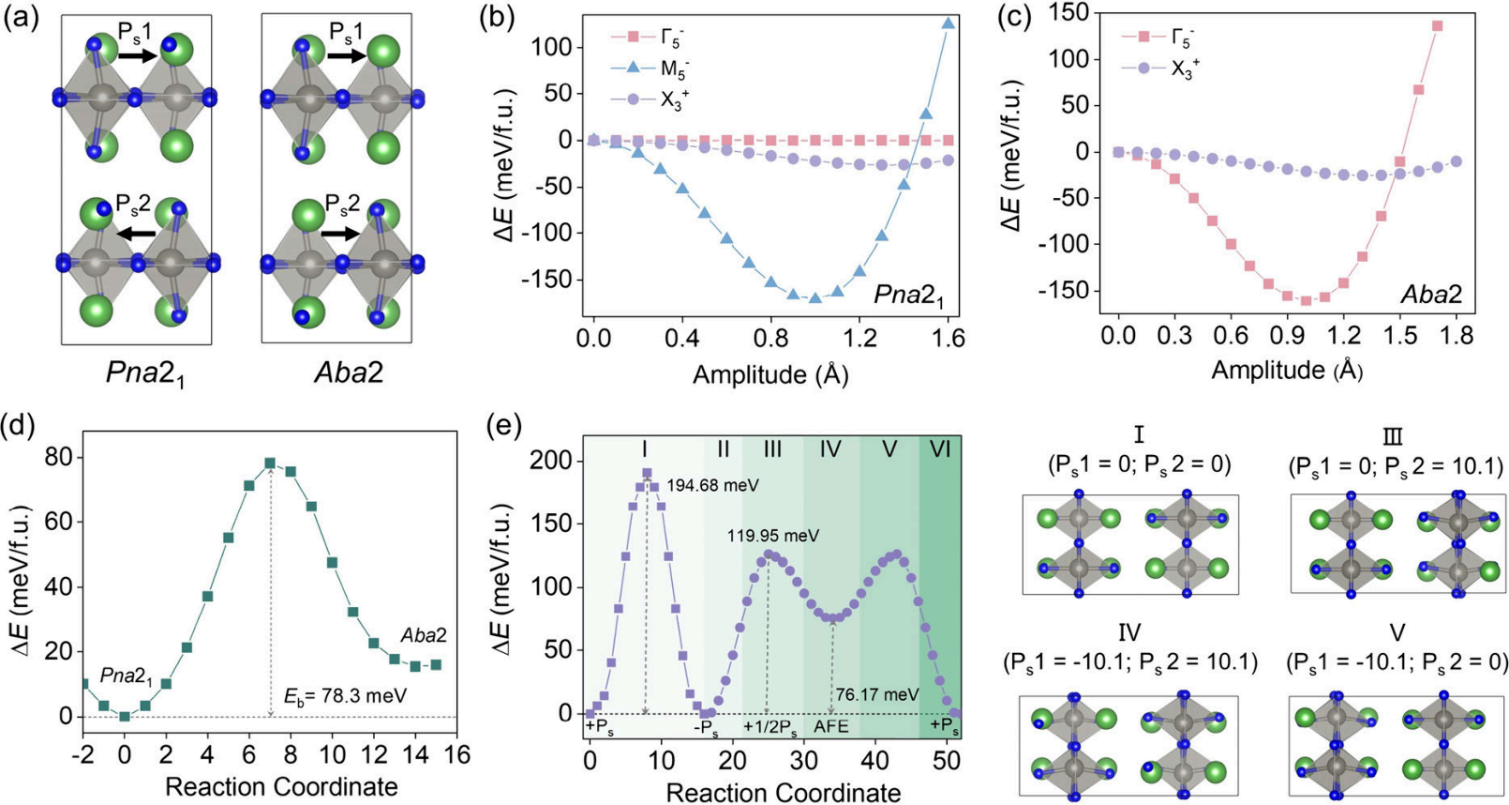
(**a**) Crystal structures of La_2_WN_4_ in the antiferroelectric *Pna*2_1_ and ferroelectric *Aba*2 phases. Black arrows indicate layer-resolved ferroelectric polarization (*P*_s_). (**b**, **c**) Energy as a function of the amplitude of the primary main distortion modes, scaled relative to their equilibrium values in the (**b**) *Pna*2_1_ phase and (**c**) *Aba*2 phase. (**d**) Minimum-energy pathways for the *Pna*2_1_ to *Aba*2 phase transition obtained from the SS-NEB calculations; *E*_b_ is the energy barrier for the phase transition. (**e**) Minimum-energy pathways obtained from the CI-NEB calculations and representative intermediate structures for switching between two ferroelectric (FE) states (FE1 to FE2) within the *Aba*2 phase, showing the polarization switching barriers along transition path 1 (I) and path 2 (II to VI). The unit of ferroelectric polarization is μC/cm^2^.

This qualitative difference in polar behaviors can be traced to distinct combinations of unstable symmetry-adapted modes condensed from the *I*4/*mmm* parent structure. We therefore conducted symmetry-mode decomposition for both polar structures with respect to *I*4/*mmm*. For the AFE *Pna*2_1_ phase, the stabilization is dominated by the M_5_^–^ mode at the Brillouin-zone boundary (M point), which primarily corresponds to the inversion rotation of adjacent octahedral layers. The X_3_^+^ mode provides a smaller energy but non-negligible FE stabilization; together, M_5_^–^ and X_3_^+^ account for 55.85% and 43.93% of the total polarization amplitude, respectively (Figure 3b and Table S3), while the Γ_5_^–^ component contributes only 0.22%. In contrast, in the FE *Aba*2 phase the energy gain is governed by the Γ_5_^–^ mode, which describes the collective polar displacements and directly generates the macroscopic polarization, whereas the X_3_^+^ mode cooperatively optimizes the octahedral tilts (Γ_5_^–^: 53.45%; X_3_^+^: 46.55%), as shown in Figure 3c and Table S3. The clear symmetry and energetic separation of these instabilities suggest that selective excitation or suppressing specific phonon modes could enable controlled interconversion between the AFE *Pna*2_1_ and FE *Aba*2 phases.

Based on this insight, we calculated the minimum energy path connecting *Pna*2_1_ and *Aba*2 using the solid-state nudged elastic band method (SS-NEB) (Figure 3d). The resulting barrier for AFE–FE interconversion is 78.3 meV/f.u., a magnitude that can be experimentally achieved,^14^ indicating that the two phases are thermodynamically interconvertible. Notably, both the FE and AFE phases remain dynamically stable at 300 and 400 K, indicating that thermal fluctuations are insufficient to induce spontaneous reversal despite the relatively low switching barrier. For device applications, an equally important figure of merit is the polarization-switching barrier within the FE *Aba*2 phase, as it governs both the intrinsic switching kinetics and the required operating voltage. Climbing image nudged elastic band (CI-NEB) calculations (Figure 3e) reveal two distinct switching pathways. Path I corresponds to a direct 180° reversal (FE–PE–FE), in which the polarization flips in a single step; this pathway exhibits a relatively high barrier of 194.68 meV/f.u. and is accompanied by an evolution from a semiconducting to a metallic state along the path (Figure S9). Path II–VI proceeds via a multistep, coordinated mechanism involving *i*) a locally polarized intermediate and *ii*) an intermediate state with vanishing macroscopic polarization, with representative stepwise barriers of 119.95 and 76.17 meV/f.u., respectively. The substantially lower overall activation energies of Paths II–VI indicate that polarization reversal is kinetically favored to occur through this multistep route. Unlike Path I, where metallicity persists over a broader region, Paths II and VI exhibit a sharp, transient metallicity localized at the transition state (Figure S10), effectively screening the electrostatic energy cost and reducing the switching barriers. As shown in Table S4, the calculated switching barrier of La_2_WN_4_ (∼78 meV/f.u.) is comparable to those reported for representative 2D ferroelectric materials. These comparisons suggest that the FE switching barrier in La_2_WN_4_ is relatively low within the family of known 2D ferroelectric systems, highlighting its distinct microscopic switching pathway and supporting its potential for low-field switching. Notably, the AFE intermediate along the switching path (space group *P*1, with an energy of 93.25 meV/f.u. higher than that of the *Pna*2_1_ phase) is distinct from the AFE phase, confirming that the latter is a true local minimum rather than a transient state.

Importantly, the transition-state configuration along the low-barrier pathway closely resembles a locally antiferroelectric configuration, dominated by the Γ_5_^–^ mode, whereas the fully polarized *Aba*2 ground state is governed by the Γ_5_^–^. Thus, polarization switching in *Aba*2 must transiently traverse an AFE-like distortion, directly linking the macroscopic switching behavior to the microscopic competition between FE (Γ_5_^–^) and AFE (M_5_^–^) order parameters. This mechanistic connection provides a microscopic basis for engineering polarization reversal by targeted control of specific phonon instabilities and their associated kinetic pathways.

The AFE–FE interconversion in La_2_WN_4_ is associated with a relatively small energy barrier, suggesting that the polarization state can be efficiently tuned by external stimuli such as epitaxial strain or an applied electric field. Figure 4a shows the total energies of the AFE *Pna*2_1_ and FE *Aba*2 phases under biaxial epitaxial strain from −5% to +5%. After full relaxation under strain, the FE and AFE phases retain the *Aba*2 and *Pna*2_1_ phases, respectively, indicating the absence of a strain-induced structural transition. Under compressive strain (−5% to – 1%), the FE *Aba*2 phase is energetically favored, whereas for strains from −1% to +5% the AFE *Pna*2_1_ phase becomes more stable. Notably, a modest compressive strain of 1% is sufficient to trigger the transition from AFE to FE, highlighting the strain sensitivity of the polarization order in La_2_WN_4_. Figure 4b further presents the spontaneous polarization and band gap of the strain-stabilized ground state as a function of the epitaxial strain. Within the FE regime (−5% to −1%), the polarization increases with increasing compression, accompanied by correlated evolution of the band gap. This trend is consistent with the strain-enhanced condensation of the polar mode (Γ_5_^–^); that is, compressive epitaxy strengthens the ferroelectric instability and amplifies the macroscopic dipole. Simultaneously, the band gap demonstrates a nearly linear dependence on strain, expanding steadily from 0.99 eV at 5% compression strain to 1.89 eV at 5% tension strain. Concomitantly, the polarization-switching barrier within the *Aba*2 phase (Figure 4c) decreases from 116.25 to 40.38 meV/f.u. as the compressive strain is tuned from −5% to −1%. This downward trend indicates that the compressive strain not only stabilizes the FE state but also facilitates polarization reversal by lowering the inversion barrier.

**Figure 4:**
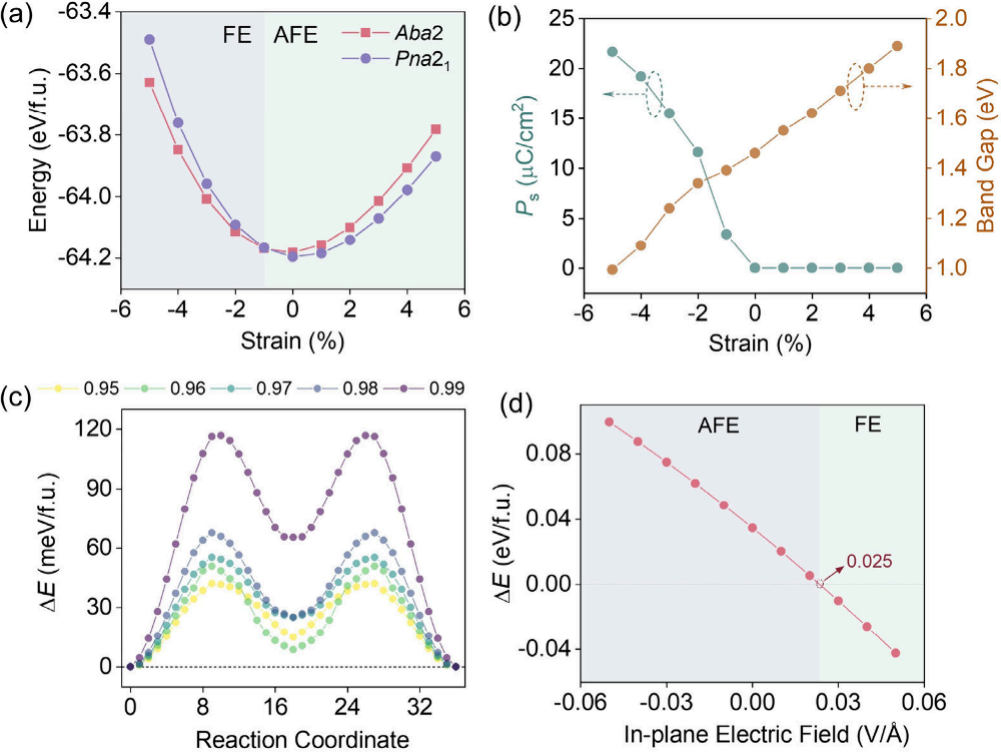
(**a**) Calculated energies of the *Pna*2_1_ and *Aba*2 phases in La_2_WN_4_ as a function of the epitaxial strain (−5% to 5%). (**b**) Strain dependence of the ferroelectric polarization and band gap of La_2_WN_4_ in its ground-state phase. (**c**) CI-NEB minimum-energy pathways for switching between two FE states (FE1 to FE2) within the *Aba*2 phase under different epitaxial strains. (**d**) Energy difference between *Pna*2_1_ and *Aba*2 phases, defined as *ΔE* = *E*(*Aba*2) – *E*(*Pna*2_1_), as a function of the applied in-plane electric field. Positive and negative fields correspond to the [100] and [1̅00] crystallographic directions, respectively.

To assess the feasibility of electric-field control, we next examined the energy competition between the AFE and the FE phases under an in-plane electric field. Figure 4d shows the change of the energy difference (Δ*E* = *E*_FE_ – *E*_AFE_), as a function of field strength and direction. A field applied along [1̅00] stabilizes the AFE phase, whereas a field as small as 0.025 V/Å along [100] is sufficient to stabilize the FE phase. The critical field is well within experimentally accessible limits,^48,49^ which demonstrates that an electric-field-driven AFE–FE transition in La_2_WN_4_ is readily achievable. Moreover, the density of states (DOS) of both the FE and AFE phases were calculated under an external electric field of 0.025 V/Å (Figure S11), and both phases remain semiconducting, with band gaps of 1.68 and 1.77 eV, respectively, indicating that the switching field does not induce field-induced metallization or electronic instability. Microscopically, the field-induced transformation corresponds to a change in the dominant distortion from M_5_^–^-type AFE modes to Γ_5_^–^-type FE modes, effectively amounting to a 180° reversal of the WN_6_ octahedral distortion. Therefore, these results establish La_2_WN_4_ as a promising platform for low-power polarization-control devices enabled by strain- and field-tunable AFE–FE switching.

Due to its intrinsically layered structure (Figure 5a), RP-type La_2_WN_4_ is expected to be exfoliated into a 2D monolayer (Figure S12). A suite of stability assessments (e.g., formation and binding energies, phonon dispersion, AIMD, and elastic constants) demonstrates that the monolayer is thermodynamically, dynamically, and mechanically stable (Figures S13–S15 and Table S5). At the HSE+U+SOC level, the 2D La_2_WN_4_ monolayer remains a semiconductor with a band gap of 1.63 eV (Figure S16). Taking the paraelectric *P*4/*mbm* phase as the reference, the fitted energy–polarization curve exhibits a typical anharmonic double-well potential (Figure S17b), confirming an intrinsic ferroelectric stability in the monolayer. Accordingly, the monolayer develops a large in-plane spontaneous polarization of 2.78 C/m. As in the bulk FE phase, the polarization mainly originates from WN_6_ octahedral distortions, together with relative displacements of N atoms. The resulting polarization is comparable to, or even exceeds, those reported for representative 2D ferroelectrics such as SnS,^50^ In_2_Se_3_,^51^ and WTe_2_.^52^

**Figure 5:**
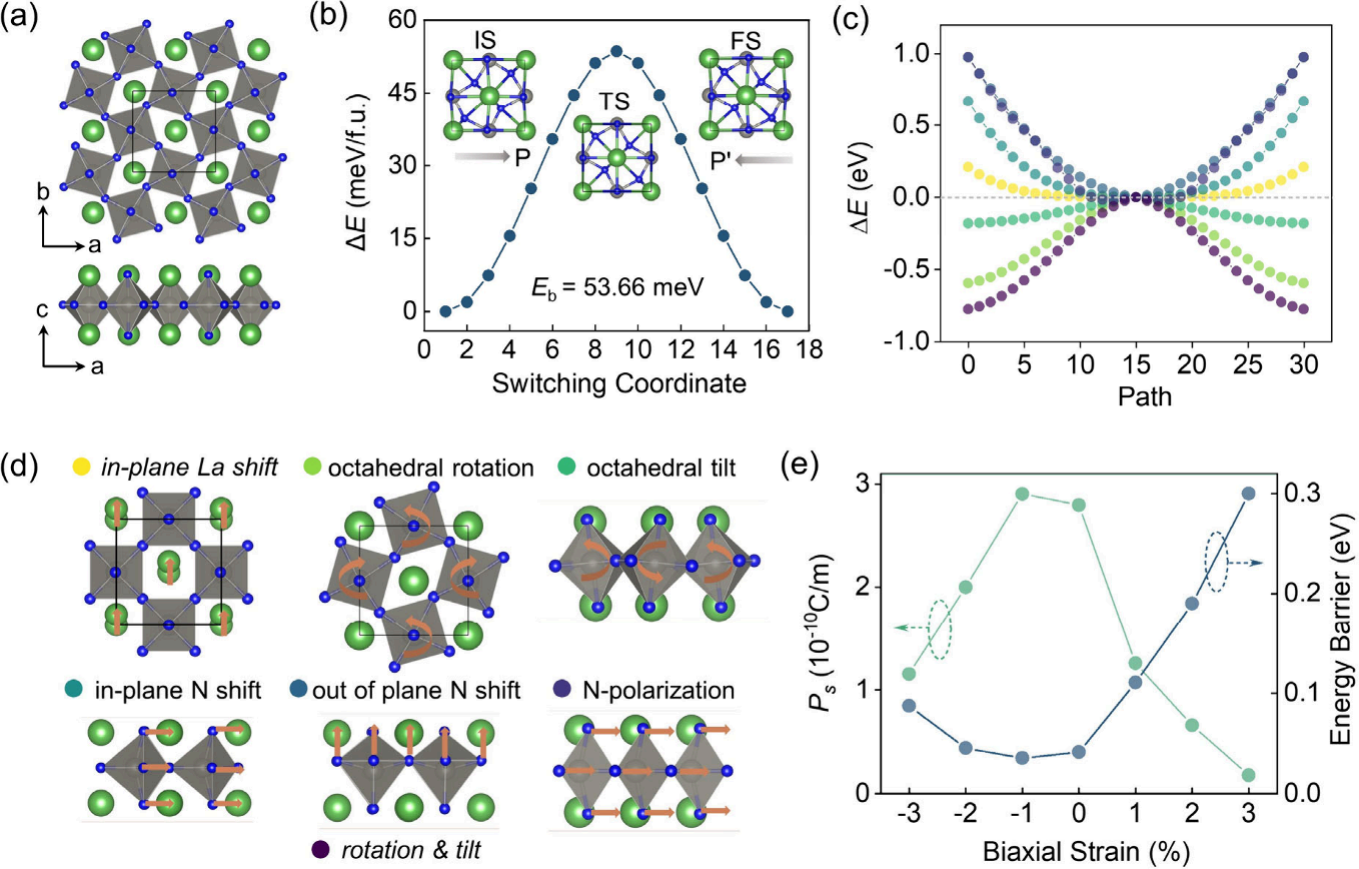
(**a**) Top and side views of the crystal structures of the La_2_WN_4_ monolayer. (**b**) Minimum-energy pathways and representative structures for ferroelectric switching in the La_2_WN_4_ monolayer along the FE1–PE–FE2 transition. IS, TS, and FS represent the initial FE1 state, the paraelectric transition state (PE), and the final FE2 state, respectively. Arrows indicate the polarization direction, and *E*_b_ is the switching barrier. (**c**) Energy evolution of representative lattice-distortion modes in a La_2_WN_4_ monolayer as the structural transition from a hypothetical high-symmetry phase. (**d**) Schematic illustrations of the considered distortion modes, including in-plane La shift, octahedral rotation, octahedral tilt, in-plane N shift, out-of-plane N shift, and N-site polarization. Curved arrows indicate octahedral rotation, and straight arrows denote ionic displacements. (**e**) Effect of biaxial strain (−3% to 3%) on the ferroelectric polarization (green lines) and switching barrier (blue lines) of the La_2_WN_4_ monolayer. The biaxial strain is applied along the *a* and *b* directions.

The switchability of this 2D ferroelectric state is further supported by CI-NEB calculations, which yield a polarization-switching barrier of 53.66 meV/f.u. (Figure 5b). This barrier is lower than those in CrCoS_4_ ^53^ and Sc_2_CO_2_ ^54^ and comparable to Cs_2_PbF_4_,^55^ indicating that polarization switching can be achieved under practical electric fields. Consistently, the energy-mode analysis, including in-plane La shift, octahedral rotation, octahedral tilt, in-plane N shift, out-of-plane N shift, and N-site polarization (Figures 5c, 5d), shows that octahedral distortions provide dominant stabilization by substantially lowering the total energy. Biaxial strain engineering (3% to +3%) further highlights the robustness and tunability of the layered ferroelectric state (Figure 5e). The system is semiconducting over the entire strain window, while the spontaneous polarization (*P*_s_) exhibits a nonmonotonic dependence on strain: *P*_s_ increases as strain is tuned from −3% to −1%, reaches a maximum near −1%, and then decreases slightly toward +3%. Importantly, the polarization direction is preserved throughout, confirming that 2D ferroelectric configuration is intrinsically stable and highly strain-tunable. We also assess the possibility of antiferroelectric phases in the monolayer via lateral displacements (Figures S18 and S19). The total energy calculations reveal that all such configurations are energetically higher than the paraelectric state, confirming that the antipolar order present in the bulk is suppressed upon exfoliation due to the loss of interlayer electrostatic coupling.

In summary, we demonstrate that the 2D RP nitride perovskite La_2_WN_4_ hosts competing FE and AFE states governed by WN_6_ octahedral distortions and polar displacements of N anions. Both polar phases are locally stable, and AFE–FE interconversion and polarization reversal within the FE state proceed through comparatively low barriers. A biaxial compressive strain of 1% or an in-plane electric field of 0.025 V/Å is sufficient to induce reversible switching between the two polar phases. This low-barrier polar response persists down to the 2D limit, where a La_2_WN_4_ monolayer retains substantial in-plane polarization together with a low switching barrier. Compared with conventional FE polarization switching, the low-barrier AFE–FE transition is more promising for devices that exploit switching between distinct antipolar and polar states, such as multistate memory concepts, energy-storage capacitors, tunable dielectric/capacitive devices, and low-power phase-switching electronics. La_2_WN_4_ therefore provides a model system in which *d* ^0^ lattice instabilities, AFE–FE mode competition, and layered-structure effects coexist, offering a realistic basis for designing and exploring low-barrier polarization functionalities in nitride perovskites.

## Supplementary Material



## Notes

Computational methods; number of *d*-electrons; PDOS; phonon dispersion curves for bulk and monolayer; AIMD simulations; projected band structures; Born effective charges and dipole moments; electron localization function; band gap variation between different phases; band gap and ferroelectric polarization calculated at the PBE and HSE level; exfoliation energies; Bader charge analysis; lattice-distortion mode decomposition; stress–strain relations; ferroelectric switching barriers; calculated elastic constants, Young’s modulus, and Poisson’s ratio (PDF)
