# Supplementary material for: Ultralow-Barrier Antiferroelectric–Ferroelectric Transition Enabled by Competing Polar Distortion in Two-Dimensional Ruddlesden–Popper Nitride Perovskites
Source: Nano Lett. 2026 Apr 8;26(15):5154–61. doi: 10.1021/acs.nanolett.6c00448 (PMC13107449; doi:10.1021/acs.nanolett.6c00448)
Supplement: Supplementary file 1 [file nl6c00448_si_001.pdf]

Supporting Information

Ultralow-Barrier Antiferroelectric–Ferroelectric Transition Enabled by Competing Polar Distortion in Two-Dimensional Ruddlesden–Popper Nitride Perovskites

Shuyi Lin[1], Qiong Lei[2,], Jun Yin[1,*]*

[1]Department of Applied Physics, Research Center for Organic Electronics, Research Center for Organic Electronics, The Hong Kong Polytechnic University, Hung Hom, Kowloon, Hong Kong 999077, China

[2]Macao Institute of Materials Science and Engineering (MIMSE), Faculty of Innovation Engineering, Macau University of Science and Technology, Taipa, Macao, 999078, China

Corresponding Authors

* qlei@must.edu.mo; jun.yin@polyu.edu.hk

Computational Methods

Density functional theory (DFT) calculations were performed using the projector augmented-wave (PAW) method as implemented in the Vienna Ab initio Simulation Package (VASP).[1] Exchange–correlation effects were treated within the generalized gradient approximation (GGA) using the Perdew-Burke-Ernzerhof (PBE) functional.[2] The core–valence interactions were described using the PAW potentials, with La ($5s^2 5p^6 5d^1 6s^2$), W ($5s^2 5p^6 5d^4 6s^2$), and N ($2s^2 2p^3$) treated as valence electrons. A plane-wave kinetic energy cutoff of 550 eV was used. Brillouin-zone sampling used a Γ-centered 9×9×1 *k*-point mesh for both structural relaxations and electronic-structure evaluations. The electronic and ionic convergence criteria were set to 10^{-5} eV for total energy and 0.01 eV/Å for residual forces, respectively.

To account for on-site electron correlation, Hubbard parameters (U_{eff}) were determined using the linear-response approach of Cococcioni (Figure S1), with U evaluated as $U = \frac{\partial V_I}{\partial N_I^{SCF}} - \frac{\partial V_I}{\partial N_I^{NSCF}}$. The resulting U_{eff} values were set to 3 eV for La-5*d* and 1 eV for W-5*d* states. More accurate electronic structures were further obtained using the hybrid Heyd-Scuseria-Ernzerhof (HSE06) with a mixing parameter of 25% and a screening parameter of 0.2 $Å^{-1}$. The plane-wave energy cutoff was set to 500 eV, and a Monkhorst-Pack *k*-point mesh of 4×4×2 was used. Spin-orbit coupling (SOC) was included in electronic-property calculations.[3]

Minimum-energy pathways and energy barriers for ferroelectric (FE) switching between symmetry-related variants in RP-type La_2WN_4 were computed using the climbing image nudged elastic band (CI-NEB) method.[4] The structures were relaxed until the forces on each image were set to 0.02 eV/Å. The lattice parameters were fixed, while the internal atomic coordinates were relaxed during the calculations. The barrier for the AFE to FE phase transition was evaluated using the solid state nudged elastic band (SS-NEB) method.[5] Both atomic positions and lattice degrees

of freedom were allowed to vary along the reaction path. Fifteen intermediate images were used, as well as the energy and force convergence threshold were set to 10^{-5} and 0.02 eV/Å, respectively. The ferroelectric polarization was calculated using the Berry-phase formalism.[6] The Born effective charges were calculated using the density functional perturbation theory (DFPT)[7] with a plane-wave cutoff energy of 550 eV, a 6×6×2 Monkhorst–Pack *k*-point mesh. The energy and force convergence criteria were set to 10^{-8} eV and 0.01 eV/Å, respectively. The phonon spectra were calculated using PHONOPY with the finite displacement method.[8] Symmetry-mode analyses were carried out using the AMPLIMODES package.[9]

Ab initio molecular dynamics (AIMD)[10] simulations were performed to assess the thermal stability of predicted structures with a total simulation time of 10 ps. Supercells of 2×2×1 (112 atoms) and 3×3×1 (126 atoms) were used for RP-type La_2WN_4 and its 2D monolayer, respectively. Temperatures of 300 K and 400 K were maintained using a Nosé–Hoover chain thermostat. For monolayer calculations, a vacuum spacing of ~20 Å was introduced to eliminate spurious interactions between periodic images. An in-plane electric field was applied by imposing a finite homogeneous field using a discretized perturbative scheme.

The elastic constants must satisfy the mechanical stability by Born criteria. For a bulk tetrahedral crystal: $C_{ii} > 0$ ($i = 1, 3, 4, 6$), $(C_{11} - C_{12}) > 0$, $(C_{11} + C_{33} - 2C_{13}) > 0$, and $[2(C_{11} + C_{12}) + C_{33} + 4C_{13}] > 0$. For 2D systems, the corresponding criteria are $C_{11}C_{22} - C_{12}^2 > 0$ and $C_{44} > 0$.

The formation energy is calculated as:

$$E_f = (E_{La_2WN_4} - 2\mu_{La} - \mu_W - 4\mu_N)/(2 + 1 + 4)$$

where $E_{La_2WN_4}$ is the total energy of the La_2WN_4 monolayer, μ_{La}, μ_W and μ_N are the chemical potential of La, W, and N referenced to their bulk phases, respectively. The formation energy of La_2WN_4 monolayer is −0.93 eV/atom, which is lower than those of InSe (−0.60 eV/atom), $MAPbI_3$

(−0.51 eV/atom), and slightly higher than MoS_2 (−1.20 eV/atom), indicating that La_2WN_4 monolayer is strongly bonded networks.

The cohesive energy (E_{coh}) provides an additional measure of the likelihood of experimental realization and is defined as:

$$E_{coh} = (E_{\mathrm{La_2WN_4}} - 2E_{\mathrm{La}} - E_{\mathrm{W}} - 4E_{\mathrm{N}})/(2+1+4)$$

where E_{La}, E_{W} and E_{N} are the total energies of isolated La, W, and N atoms, respectively. The cohesive energy of La_2WN_4 monolayer is −8.37 eV/atom, which is lower than those of several experimentally synthesized 2D materials, such as MoS_2 (−5.02 eV/atom), germanene (−3.24 eV/atom), silicene (−3.91 eV/atom) and graphene (−7.85 eV/atom), indicating favorable energetic stability and potential synthesizability.

To evaluate the thermodynamic stability of La_2WN_4 relative to competing binary nitrides, we calculated the formation enthalpy:

$$\Delta H = E_{\mathrm{La_2WN_4}} - 2E_{\mathrm{LaN}} - E_{\mathrm{WN_2}}$$

where $E_{\mathrm{La_2WN_4}}$ is the total energy of the fully relaxed La_2WN_4 in either the $Pna2_1$ or $Aba2$ structure, E_{LaN} and $E_{\mathrm{WN_2}}$ are the energies of the most stable binary nitride phases of LaN and WN_2. The negative values (−2.12 and −2.10 eV/f.u. for $Pna2_1$ and $Aba2$, respectively) demonstrate that La_2WN_4 is thermodynamically stable with respect to binary nitrides, making it a viable target for synthesis.

The direction-dependent Young's modulus $E(\theta)$ and Possion's ratio $\nu(\theta)$ of the La_2WN_4 monolayer along an in-plane direction θ (measured from the positive x axis) are given by[11]

$$E(\theta) = \frac{C_{11}C_{22}-{C_{12}}^2}{C_{11}sin\theta^4+C_{22}cos\theta^4+(\frac{C_{11}C_{22}-{C_{12}}^2}{C_{66}}-2C_{12})cos\theta^2 sin\theta^2} \quad (3)$$

$$\nu(\theta) = \frac{(C_{11}+C_{22}-\frac{C_{11}C_{22}-C_{12}^2}{C_{66}})cos\theta^2 sin\theta^2 - C_{12}(sin\theta^4+cos\theta^4)}{C_{11}sin\theta^4+C_{22}cos\theta^4+(\frac{C_{11}C_{22}-C_{12}^2}{C_{66}}-2C_{12})cos\theta^2 sin\theta^2} \quad (4)$$

Using the calculated elastic constants, $E(\theta)$ and $\nu(\theta)$ were evaluated to quantify in-plane mechanical anisotropy. The maximum Young's modulus values of the La_2WN_4 monolayer is 161 N/m along y direction, which is less than those of graphene (342 N/m) and h-BN (275.8 N/m),[12] but considerably larger than those of silicene (62 N/m),[13] phosphorene (91.3 N/m),[14] and MoS_2 monolayer (129 N/m).[15] The Poisson's ratio exhibits only modest anisotropy, with values of 0.16 and 0.27 along the x and y directions, respectively. These values comparable to that of graphene (~0.173), indicating limited transverse contraction when the sheet is stretched along in-plane direction.

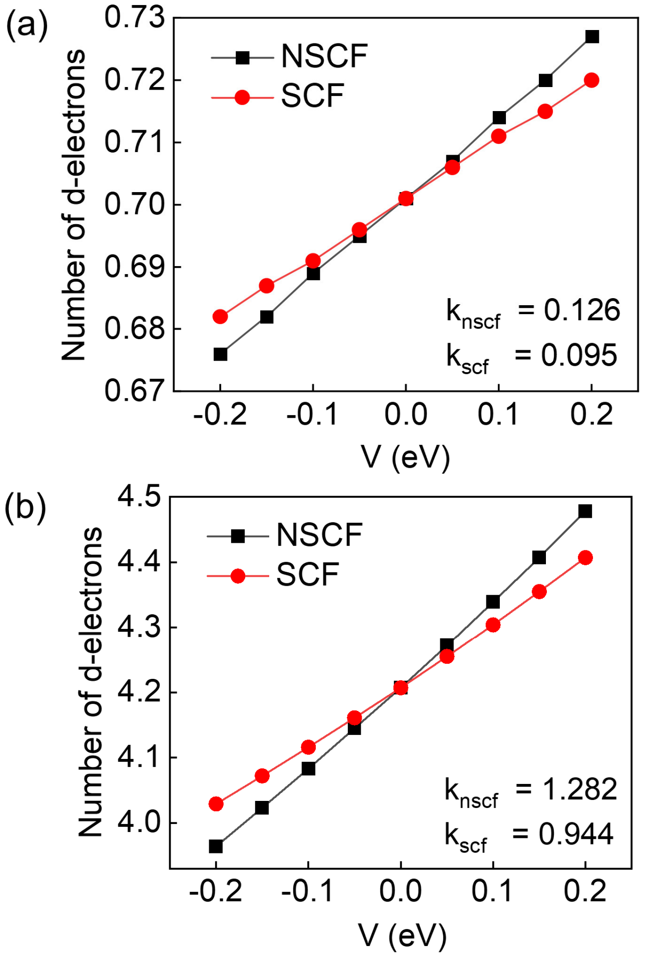

Figure S1. Linear fitting of the number of *d*-electrons as a function of the applied potential *V* for (**a**) La and (**b**) W atoms, used to determine the Hubbard U values for La and W atoms.

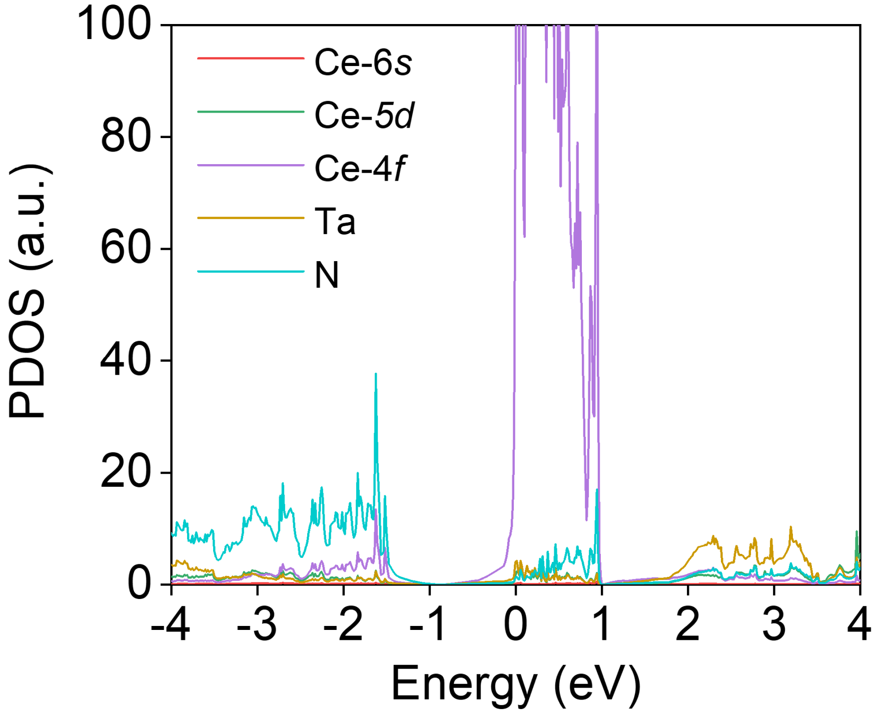

Figure S2. The projected density of state (PDOS) of Ce_2TaN_4.

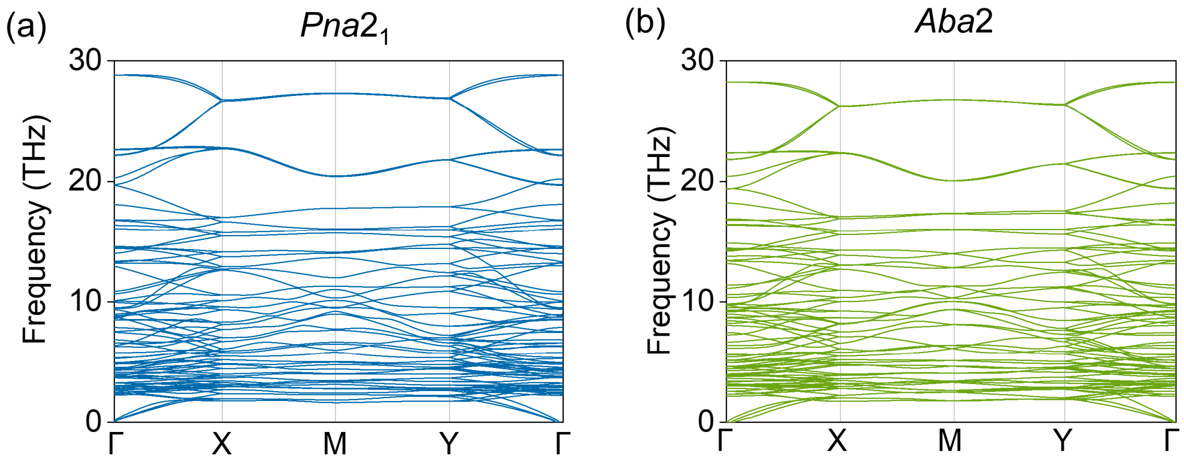

Figure S3. Calculated phonon dispersion curves in space group of (**a**) *Pna*2$_1$ and (**b**) *Aba2*. The phonon dispersions are plotted along high-symmetry paths in the Brillouin zone: Γ (0 0 0) – X (0.5 0 0) – M (0.5 0.5 0) – Y (0 0.5 0) – Γ(0 0 0). Compared with the phonon curves of the *I4/mmm* phase, the structures eliminate the imaginary frequencies.

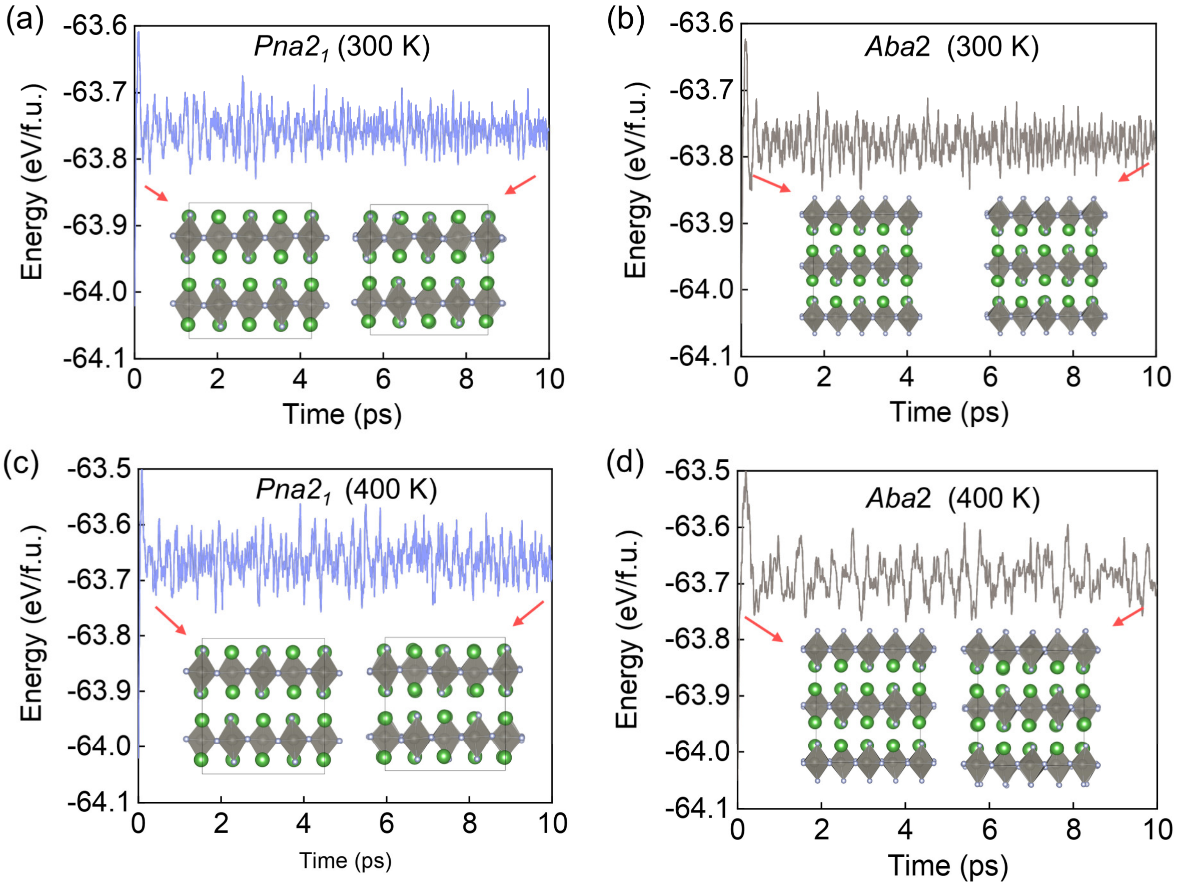

Figure S4. Evolution of total energies during AIMD simulations at (**a**, **b**) 300 K and (**c**, **d**) 400 K for the *Pna*2_1 and *Aba2* phases. Insets show the atomic structures at 0 and 10 ps, respectively.

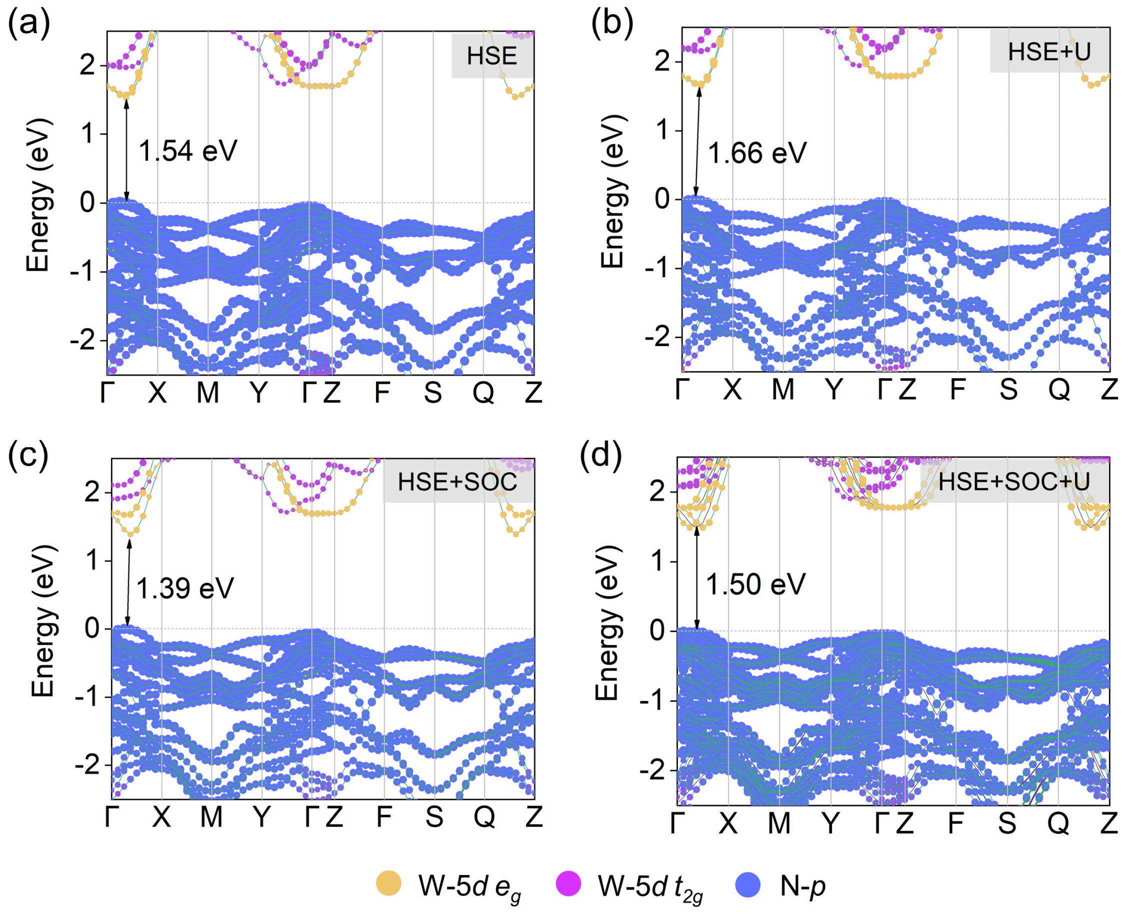

Figure S5. The projection band structures of the *Pna*2_1 in La_2WN_4 calculated using (**a**) HSE, (**b**) HSE with the Hubbard U correction, (**c**) HSE with SOC and (d) HSE with SOC and Hubbard U correction. The Fermi levels are set to zero.

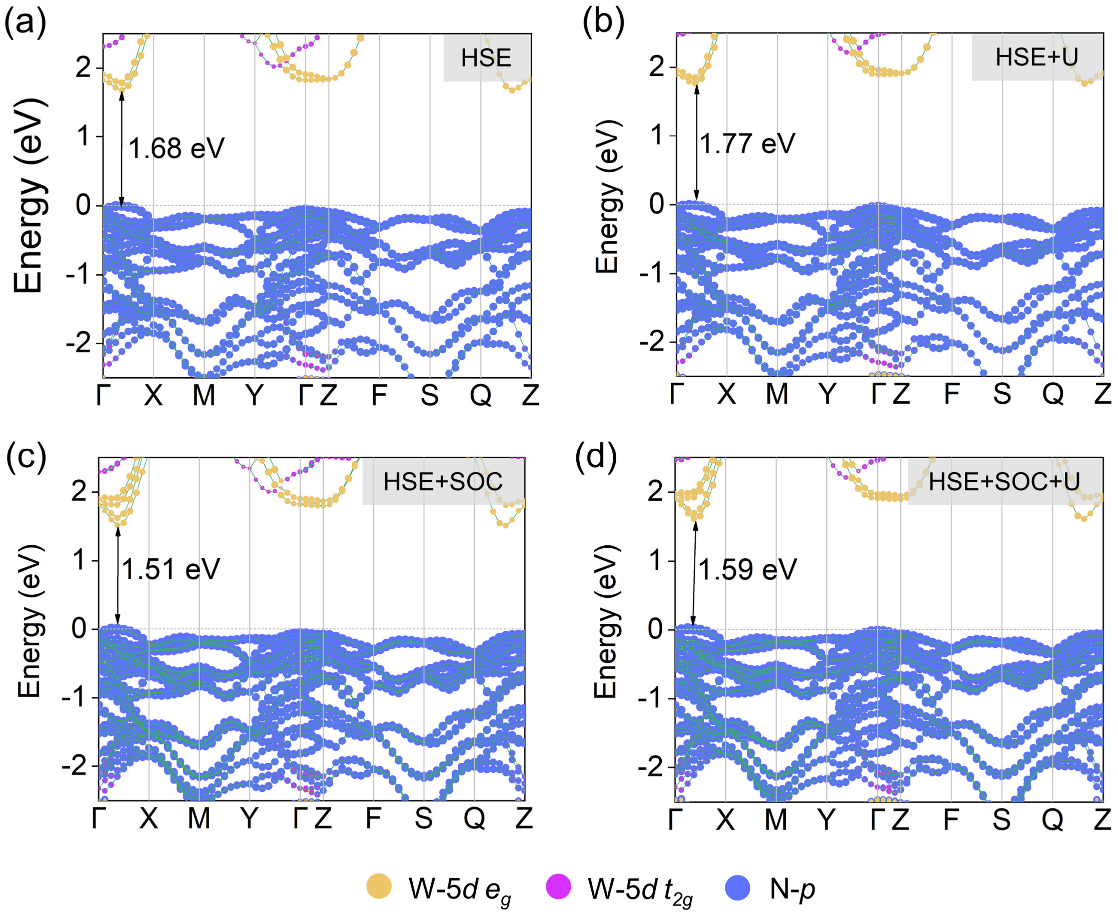

Figure S6. The projection band structures of the *Aba2* in La_2WN_4 calculated using (**a**) HSE, (**b**) HSE with the Hubbard U correction, (**c**) HSE with SOC and (d) HSE with SOC and Hubbard U correction. The Fermi levels are set to zero.

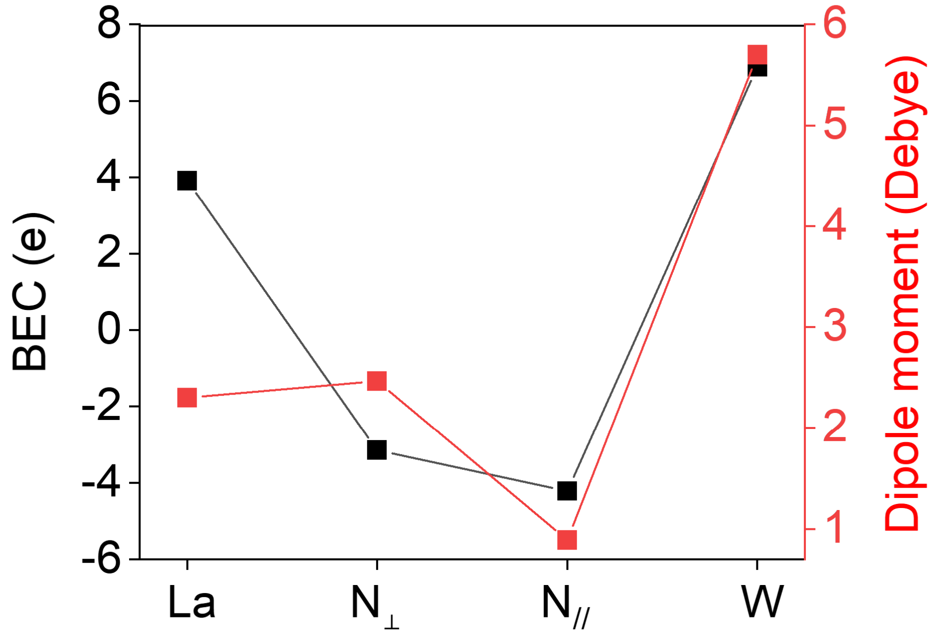

Figure S7. Born effective charges (BEC) and dipole moments for each atom of the *Aba2* in La_2WN_4. The dipole moment is calculated as the $V_{BEC}\times\Delta d$, where V_{BEC} is the Born effective charge and Δd is the atomic displacement (in Å) from the ferroelectric phase to the paraelectric phase.

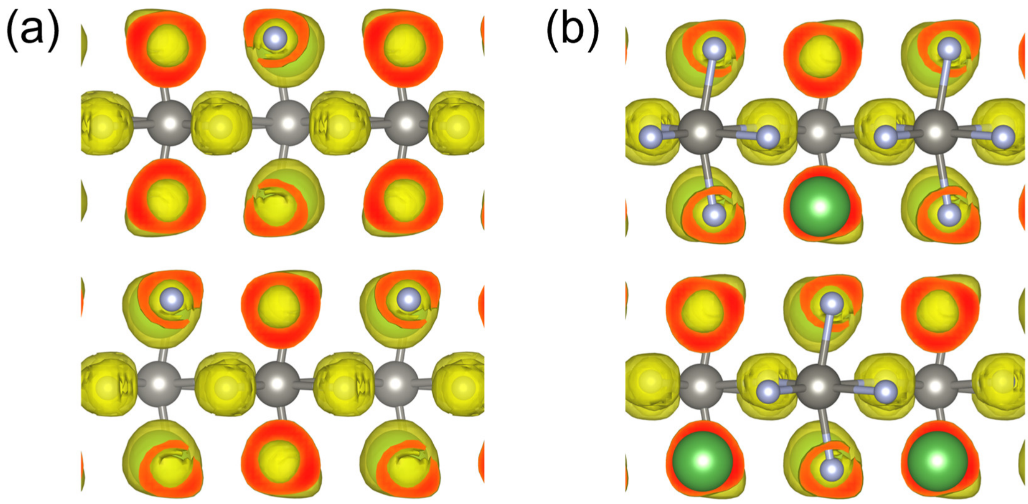

Figure S8. Three-dimensional electron localization function (ELF) isosurfaces of La_2WN_4 in the (a) *Pna*2$_1$ and (b) *Aba*2 phases plotted at an isovalue of 0.8.

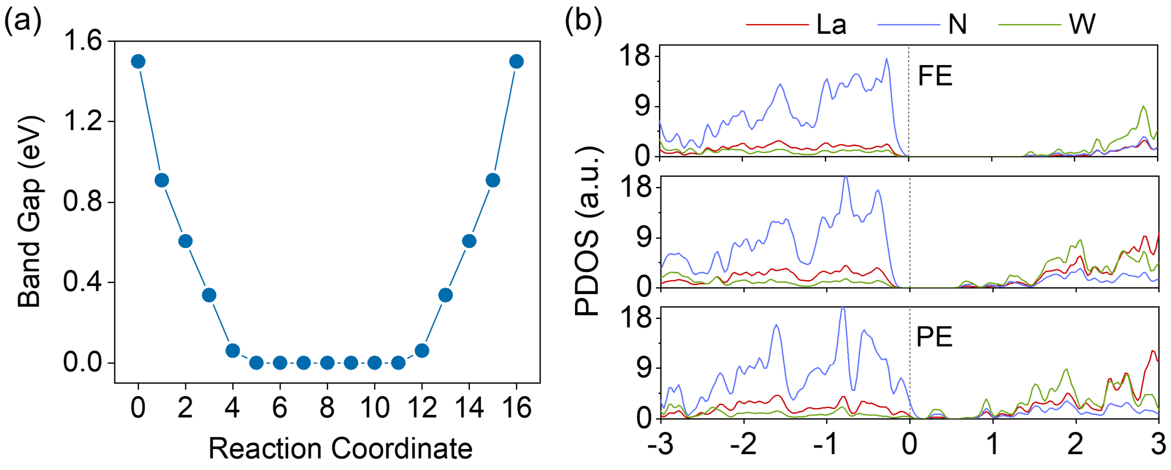

Figure S9. (**a**) Band gap variation from the FE phase1 to FE phase 2 in FE *Aba2* phase along Path I. (**b**) PDOS of *Aba2* phase in FE, intermediate and PE states. The Fermi levels are set to zero.

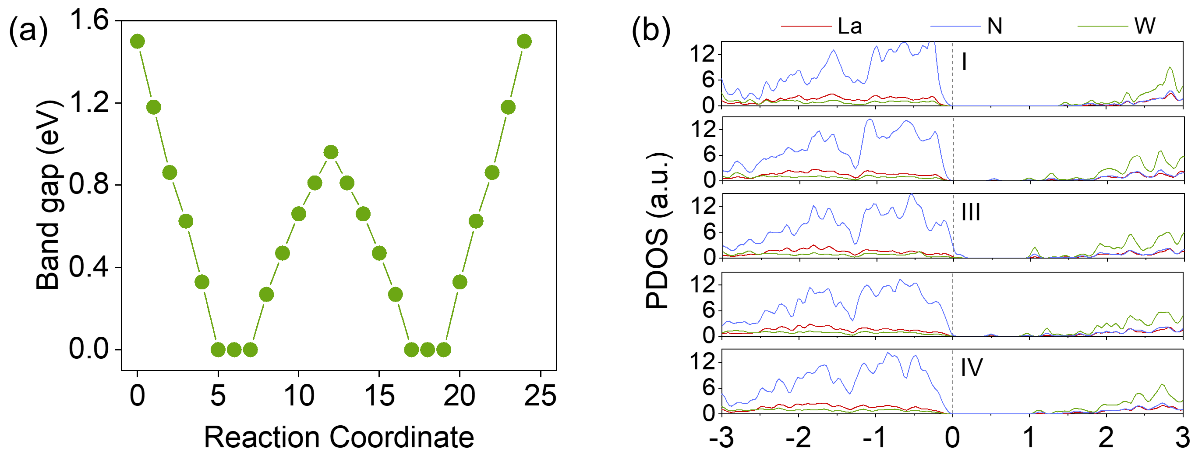

Figure S10. (a) Band gap variation from the FE phase 1 to FE phase 2 in FE *Aba*2 phase along Path II-VI . (b) PDOS of *Aba*2 phase in FE, intermediate and AFE states. The Fermi levels are set to zero.

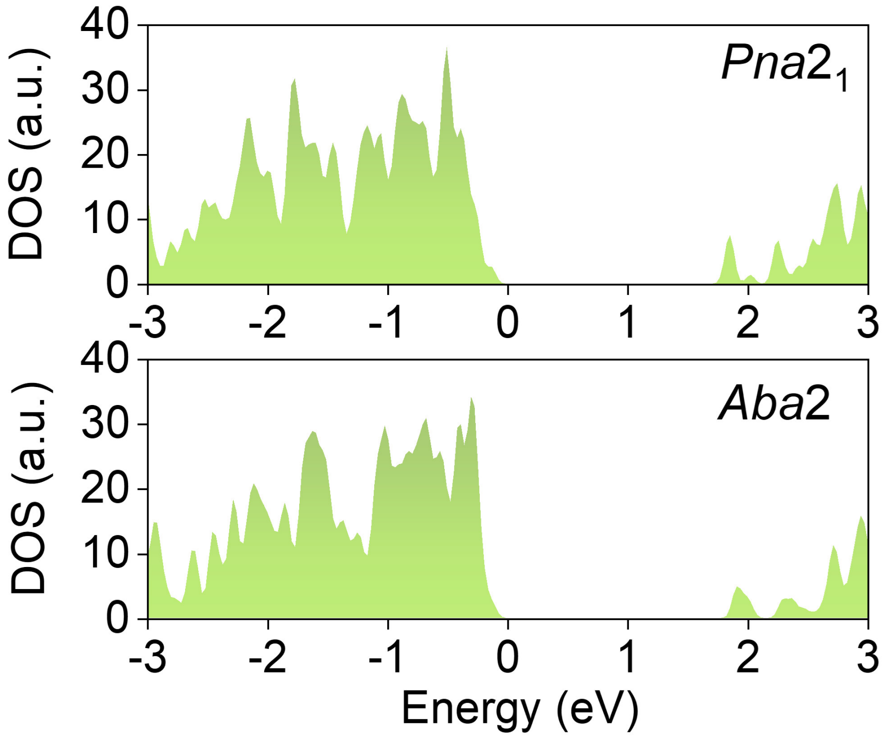

Figure S11. The density of state (DOS) of *Pna*2_1 and *Aba*2 phases for La_2WN_4 under external electric field of 0.025 V/Å.

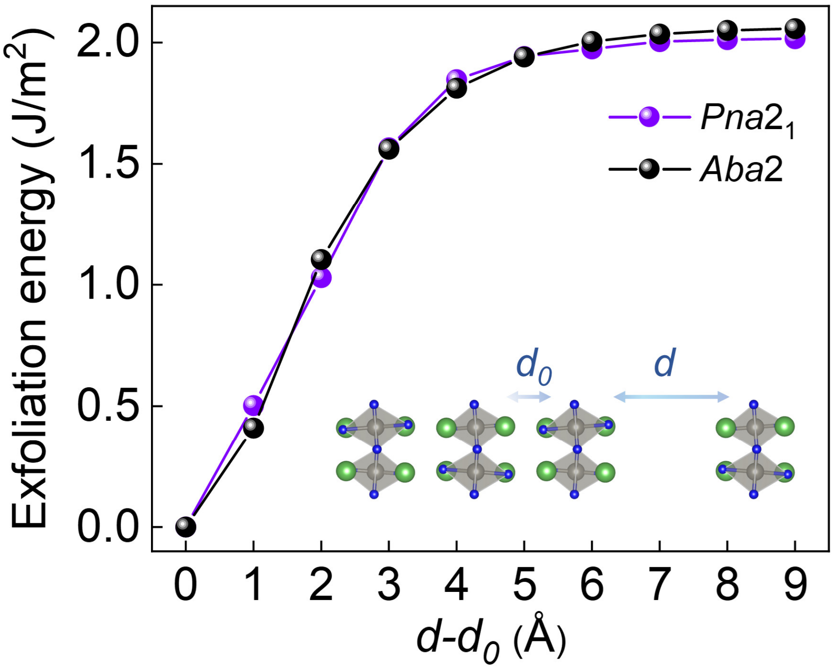

Figure S12. Exfoliation energies of La_2WN_4 monolayer from *Pna*2_1 and *Aba*2 phase. The inset is the schematic diagram of stripping monolayer structure from bulk La_2WN_4.

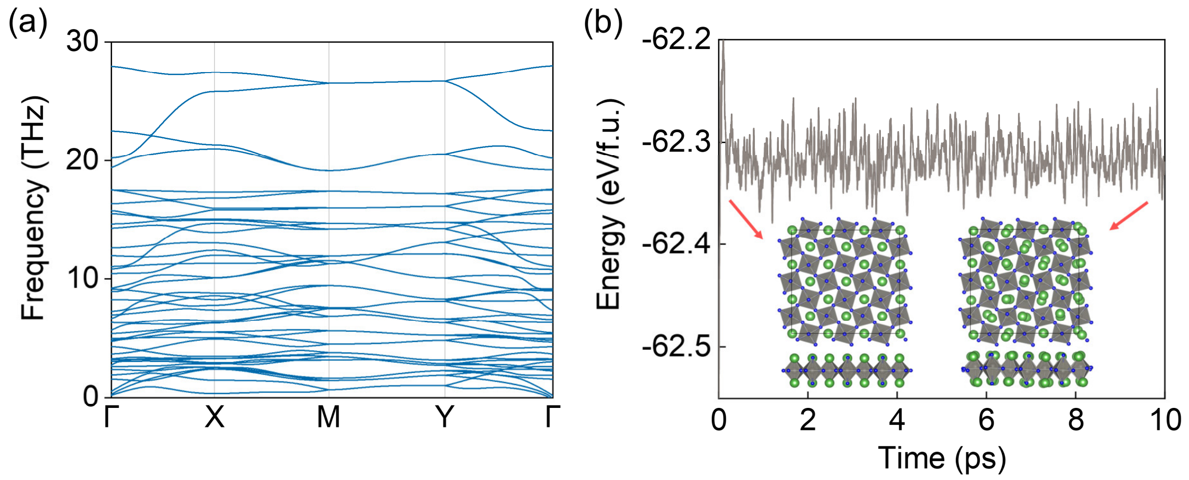

Figure S13. (**a**) Calculated phonon dispersion curves for La_2WN_4 monolayer. The phonon dispersions are plotted along high-symmetry path in the Brillouin zone: Γ (0 0 0) – X (0.5 0 0) – M (0.5 0.5 0) – Y (0 0.5 0) – Γ (0 0 0). (**b**) Evolution of total energies during AIMD simulation at 300 K for La_2WN_4 monolayer. Insets show the atomic structures at the beginning and end of 10 ps simulation. AIMD simulations have been performed at temperatures of 300 K with a time length of 10 ps. Phonon calculations show that there are no virtual frequencies throughout the Brillouin zone, proving that the structure is dynamically stable. AIMD calculation shows that La_2WN_4 monolayers can retain their structural integrity without relevant distortions, which is confirmed thermodynamic stability.

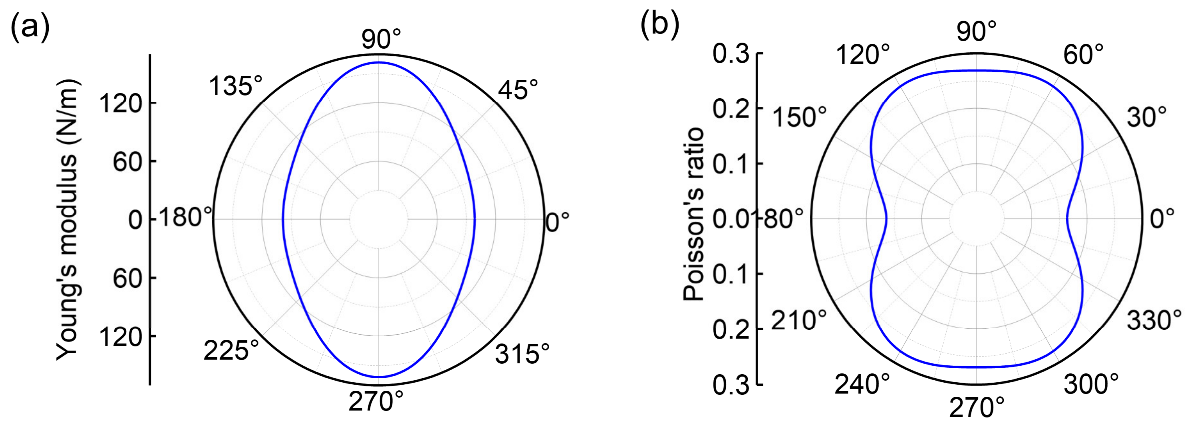

Figure S14. Polar diagrams for the (**a**) Young's modulus and (**b**) Poisson's ratio of La_2WN_4 monolayer.

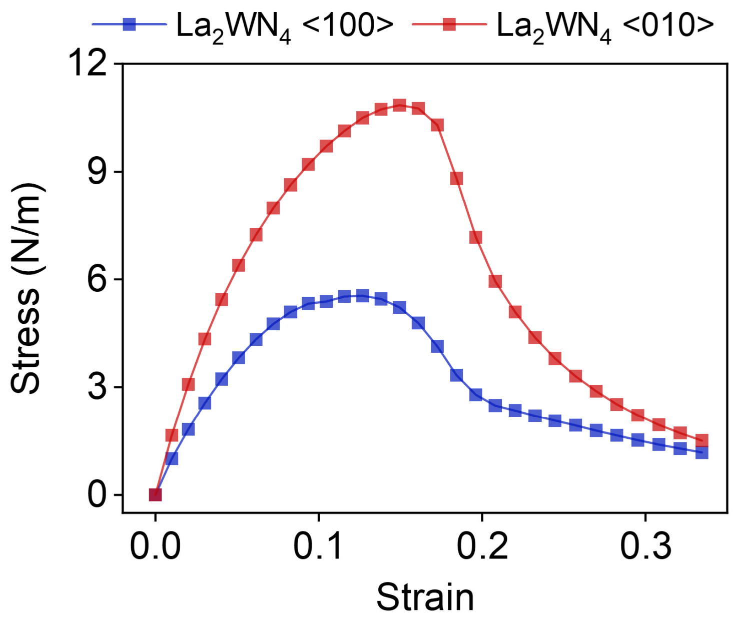

Figure S15. Calculated stress-strain relations for La_2WN_4 monolayer along <100> and <010> directions.

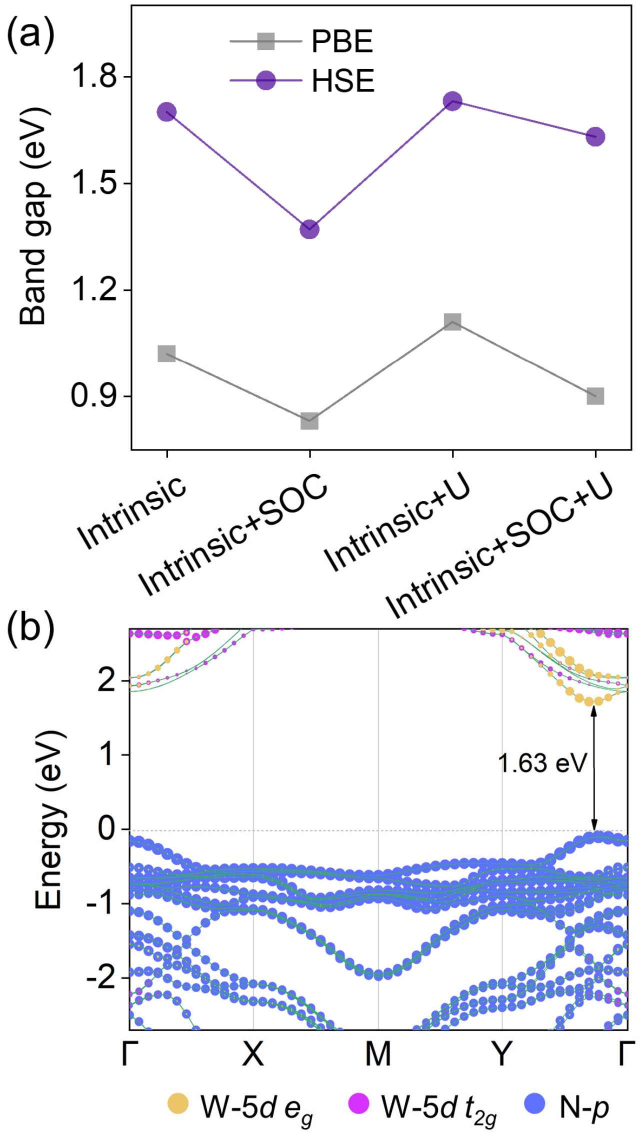

Figure S16. (**a**) The band gap of La_2WN_4 under PBE and HSE functionals with the consideration of SOC effects and the Hubbard U correction. (b)The projection band structures for La_2WN_4 monolayer. The Fermi levels are set to zero. The high symmetry points: Γ (0 0 0), X (0.5 0 0), M (0.5 0.5 0), Y (0 0.5 0), Γ (0 0 0).

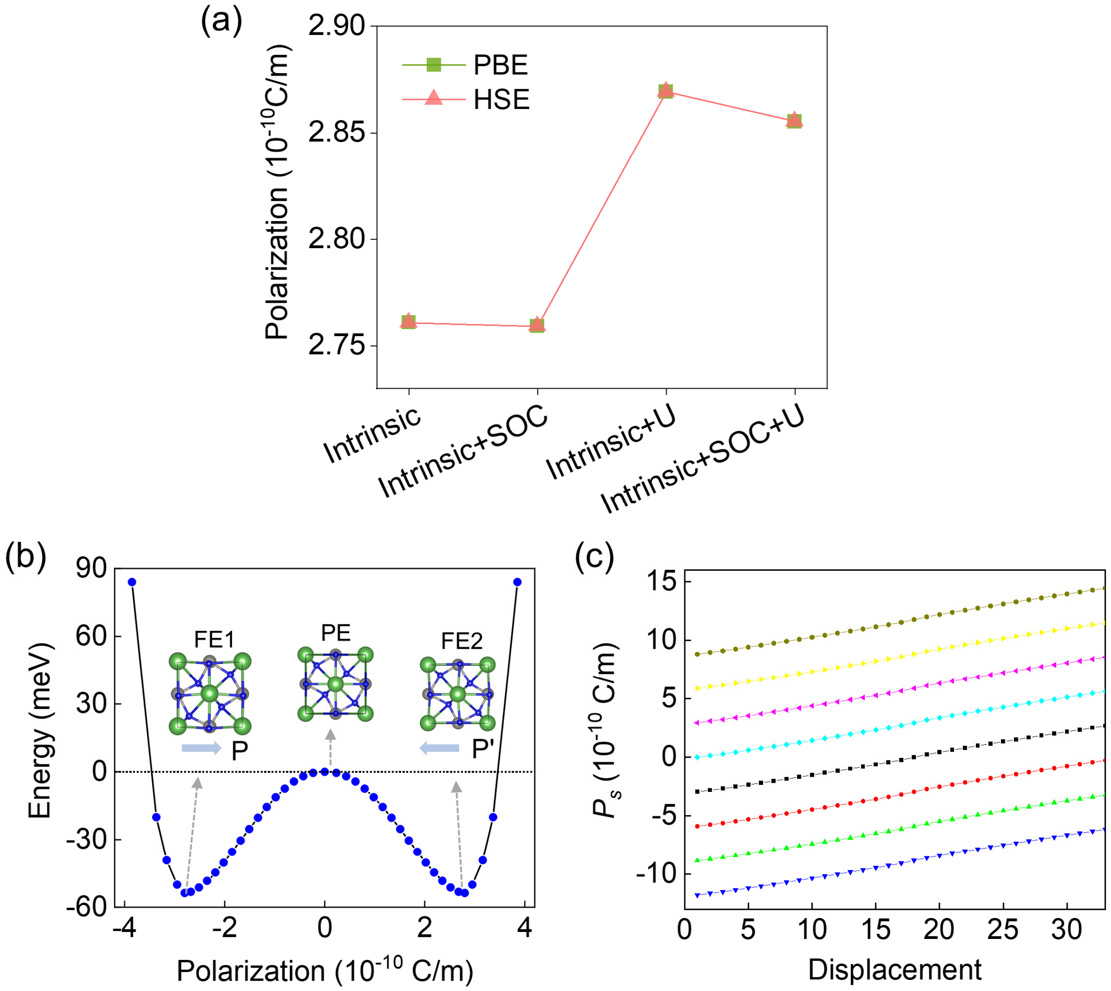

Figure S17. (**a**) Ferroelectric polarization of La_2WN_4 calculated at PBE and HSE level, incorporating SOC and the Hubbard U correction. (**b**) Double-well potential as a function of polarization for La_2WN_4 monolayer. FE1, PE, and FE2 represent the initial ferroelectric state, the paraelectric state, and the final ferroelectric state. Blue arrows indicate the direction of polarization. (**c**) Polarization corresponding to different polarization quantum of La_2WN_4. The adjacent lines correspond to a difference of one polarization quantum.

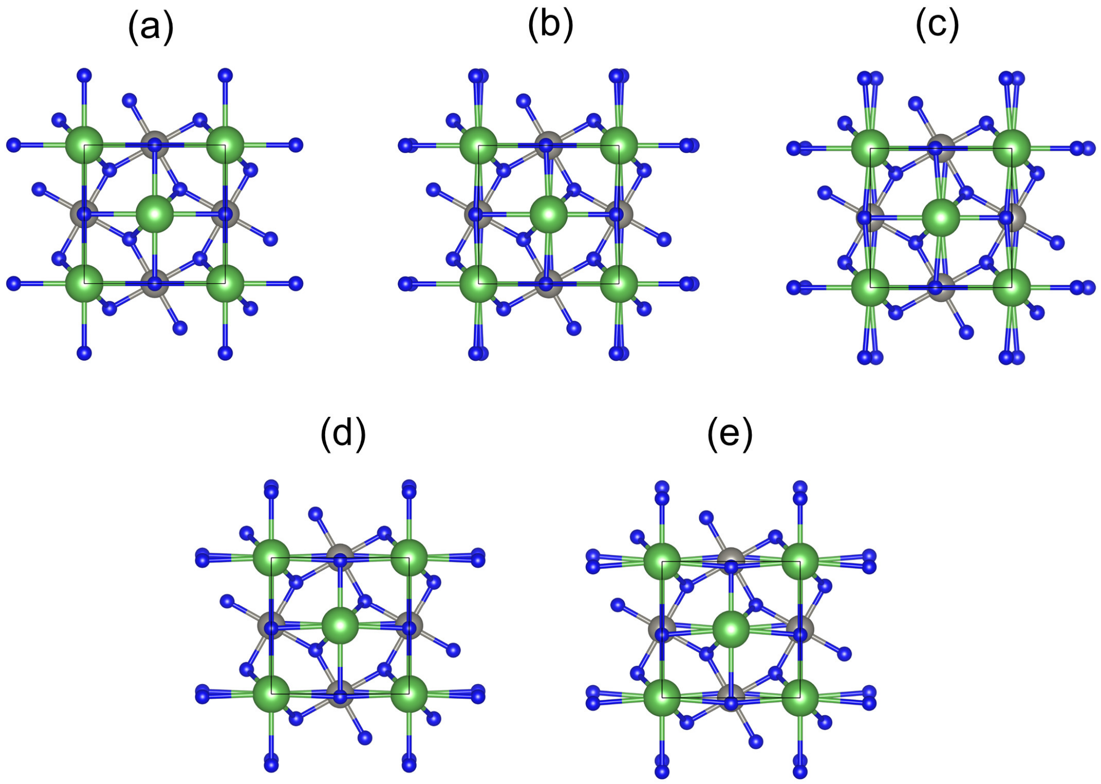

Figure S18. (**a**) Crystal structures of the paraelectric La_2WN_4 monolayer. Schematic illustrations of the antiferroelectric monolayer configurations generated by rigid displacements along the (**b**, **c**) x-axis and (**d**, **e**) y-axis.

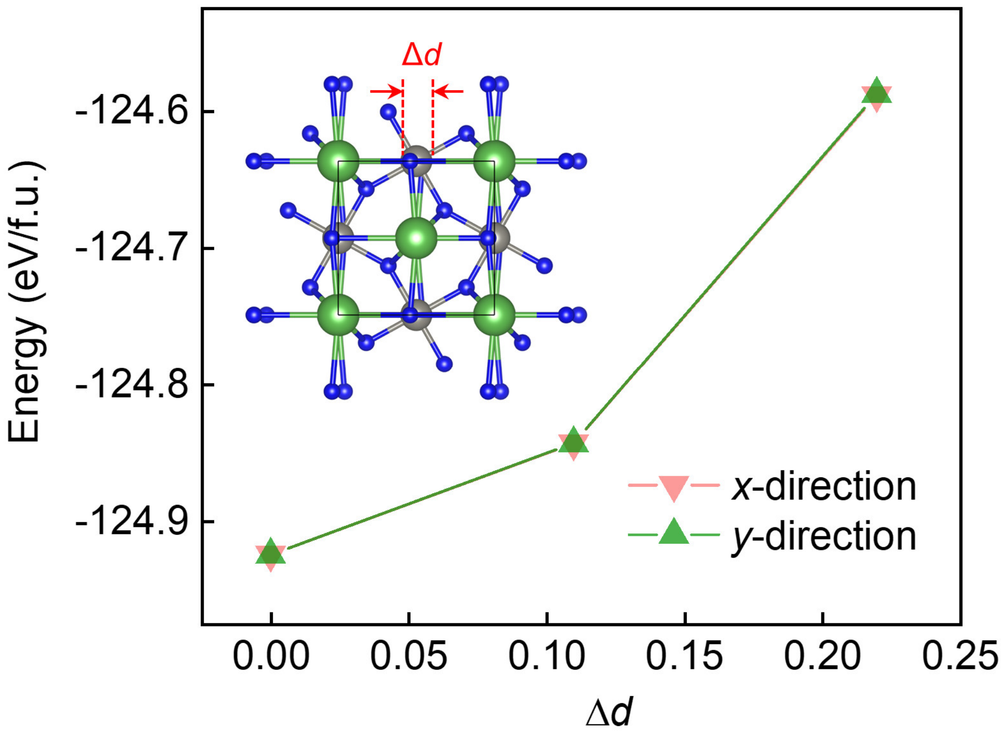

Figure S19. Energy differences of antiferroelectric configurations as a function of atomic displacement. Δ*d* represents the displacement distance of the N atom from its paraelectric phase.

Table S1. Elastic constants c_{ij} (GPa) of La_2WN_4 with space group of *Pna*2₁ and *Aba*2.

	***Pna*2₁**	***Aba*2**
c_{11}	250.22	233.88
c_{22}	230.29	277.78
c_{33}	286.12	229.83
c_{44}	63.50	92.51
c_{55}	86.27	83.93
c_{66}	90.96	61.35
c_{12}	74.27	134.97
c_{13}	128.00	60.49
c_{23}	102.20	115.47

Table S2. Bader charge analysis of La_2WN_4 for both *Pna*2$_1$ and *Aba*2 phases.

	La	W	N1	N2
*Pna*2$_1$	-1.85	-2.39	1.58	1.47
*Aba*2	-1.85	-2.38	1.57	1.47

Table S3. Symmetry-adapted lattice-distortion mode decomposition of the *Pna*2$_1$ and *Aba*2 phases of La_2WN_4.

Phases		G_5^-	X_3^+	M_5^-
*Pna*2$_1$	Amplitude (Å)	0.0027	0.5413	0.6881
	Percentage (%)	0.22	43.93	55.85
*Aba*2	Amplitude (Å)	0.4250	0.3702	0
	Percentage (%)	53.45	46.55	0

Table S4. Ferroelectric switching barrier and mechanism of reported representative 2D ferroelectric materials.

Materials	Switching barrier	Switching Mechanism	References
La_2WN_4	78 meV	AFE-FE phase transition	This work
Pr_2ReN_4	/	Metallic	*Nat. Chem.* 16, 1723 (2024)
BA_2PbCl_4	/	Cooperative coupling between A-site organic molecules and B-site inorganic	*Nano Lett*. 21, 3170, (2021)
In_2Se_3	66 meV	Interlayer sliding and atomic displacements	*ACS Nano,* 18, 26103, (2024)
WTe_2	0.6 meV	Sliding ferroelectric	*J. Phys. Chem. Lett.* 9, 7160, (2018)
MoS_2	98 meV	Sliding ferroelectric	*ACS Nano,* 18, 30360, (2024)
$CuInP_2S_6$	110.0 meV	Cu^{2+} ions displacement	*Phy. Rew. B*, 96, 235420 (2017)
SnS	39.2 meV	Lone pair driven	*Nano Lett*.16, 3236, (2016)

Table S5. Elastic constants C_{ij} (N/m), Young's modulus E (N/m), and Poisson's ratio ν of La_2WN_4 and other typical 2D monolayers.

	C_{11}	C_{22}	C_{12}	C_{44}	E_x	E_y	ν_x	ν_y
La_2WN_4	102.94	169.51	27.68	45.28	98.42	162.06	0.16	0.268
Borophene	398	170	-7	94	398	170	-0.04	-0.02
δ-phosphorene	88.64	149.21	-23.71	24.58	84.87	142.86	-0.158	-0.267
Penta-graphene	265	265	-18		263.8	263.8	-0.068	-0.068
germanene	47.3	47.3	16.7	15.3	41.4	41.4	0.35	0.35
Silicene	68.9	68.9	23.3	22.8	61	61	0.33	0.33

References

(1) Kresse, G.; Furthmüller, J. Efficient iterative schemes for ab initio total-energy calculations using a plane-wave basis set. *Phys. Rev. B* **1996**, *54* (16), 11169–11186.

(2) Perdew, J. P.; Burke, K.; Ernzerhof, M. Generalized Gradient Approximation Made Simple. *Phys. Rev. Lett.* **1996**, *77* (18), 3865–3868.

(3) Gmitra, M.; Konschuh, S.; Ertler, C.; Ambrosch-Draxl, C.; Fabian, J. Band-structure topologies of graphene: Spin-orbit coupling effects from first principles. *Phy. Rev. B* **2009**, *80* (23), 235431.

(4) Henkelman, G.; Uberuaga, B. P.; Jónsson, H. A climbing image nudged elastic band method for finding saddle points and minimum energy paths. *J. Chem. Phys.* **2000**, *113* (22), 9901–9904.

(5) Sheppard, D.; Xiao, P.; Chemelewski, W.; Johnson, D. D.; Henkelman, G. A generalized solid-state nudged elastic band method. *J. Chem. Phys.* **2012**, *136* (7), 074103.

(6) King-Smith, R. D.; Vanderbilt, D. Theory of polarization of crystalline solids. *Phys. Rev. B* **1993**, *47* (3), 1651–1654.

(7) Zhong, W.; King-Smith, R. D.; Vanderbilt, D. Giant LO-TO splittings in perovskite ferroelectrics. *Phys. Rev. Lett.* **1994**, *72* (22), 3618–3621.

(8) Parlinski, K.; Li, Z. Q.; Kawazoe, Y. First-Principles Determination of the Soft Mode in Cubic ZrO_2. *Phys. Rev. Lett.* **1997**, *78* (21), 4063–4066.

(9) Orobengoa, D.; Capillas, C.; Aroyo, M. I.; Perez-Mato, J. M. AMPLIMODES: symmetry-mode analysis on the Bilbao Crystallographic Server. *J. Appl. Crystallogr.* **2009**, *42* (5), 820–833.

(10) Parrinello, M.; Rahman, A. Crystal Structure and Pair Potentials: A Molecular-Dynamics Study. *Phys. Rev. Lett.* **1980**, *45* (14), 1196–1199.

(11) Cadelano, E.; Palla, P. L.; Giordano, S.; Colombo, L. Elastic properties of hydrogenated graphene. *Phy. Rev. B* **2010**, *82* (23), 235414.

(12) Lee, C.; Wei, X.; Kysar, J. W.; Hone, J. Measurement of the Elastic Properties and Intrinsic Strength of Monolayer Graphene. *Science* **2008**, *321* (5887), 385–388.

(13) Zhang, H.; Wang, R. The stability and the nonlinear elasticity of 2D hexagonal structures of Si and Ge from first-principles calculations. *Physica B: Condens. Matter* **2011**, *406* (21), 4080–4084.

(14) Wei, Q.; Peng, X. Superior mechanical flexibility of phosphorene and few-layer black phosphorus. *Appl. Phys. Lett.* **2014**, *104* (25), 251915.

(15) Liu, K.; Yan, Q.; Chen, M.; Fan, W.; Sun, Y.; Suh, J.; Fu, D.; Lee, S.; Zhou, J.; Tongay, S.; et al. Elastic properties of chemical-vapor-deposited monolayer MoS_2, WS_2, and their bilayer heterostructures. *Nano Lett.* **2014**, *14* (9), 5097–5103.
